# Dental health as a determinant of operational readiness in military populations: Evidence from Ceuta (Spain)

**DOI:** 10.1097/MD.0000000000046380

**Published:** 2025-12-12

**Authors:** Alejandro Bel-Blesa, Marta Hernández-Donadeu, Javier Flores-Fraile, Juan Gómez-Salgado, Luis El Khoury-Moreno, Julio Torrejón-Martínez, Eva Rosel-Gallardo, David Ribas-Pérez, Antonio Castaño-Séiquer

**Affiliations:** a Ministerio de Defensa de España, Madrid, España; b Instituto de Gestión Sanitaria, Melilla, España; c Department of Surgery, Faculty of Medicine, Universidad de Salamanca, Salamanca, España; d Department of Sociology, Social Work and Public Health. Faculty of Labour Sciences, Universidad de Huelva, España; e Safety and Health Postgraduate Program. Universidad Espiritu Santo, Guayaquil, Ecuador.; f Department of Stomatology, Faculty of Odontology, Universidad de Sevilla, Sevilla, España; g Research Group CTS503: Dental Public Health, Universidad de Granada, Granada, España.

**Keywords:** dental fitness, military personnel, oral health, periodontal disease, preventive dentistry

## Abstract

The Spanish Armed Forces require optimal oral health to ensure operability during international missions. In a cross-sectional study with a sample of 691 military personnel, 17.66% were classified as temporarily unfit (NAT), predominantly among enlisted ranks (21.7%). Employment status and smoking showed significant associations with NAT (*P* < .000), while gender, age, and alcohol consumption were not significant factors. The decayed, missing filled teeth index, particularly its decayed component, was strongly related to NAT (*P* = .000). Periodontal disease presence and the need for multiple dental treatments significantly increased NAT prevalence (*P* < .000). Use of dental floss correlated with higher fitness, whereas mouthwash use was associated with increased NAT (*P* < .05). These findings highlight the importance of prevention and risk factor control to maintain oral fitness and ensure military personnel effectiveness during deployments.

## 1. Introduction

The Spanish Armed Forces have undergone a radical change in the last century. They have gone from being Armed Forces confined to the national territory itself to being Armed Forces committed to the International Organizations of which they form part, participating in Missions and Operations outside the National Territory.^[1]^

The nature of the operations carried out by military personnel, particularly in international contexts, requires an optimum state of health that minimizes the risk of functional incapacity. For this reason, minimum medical fitness criteria are established, with special emphasis on oral health, in order to prevent common pathologies from interfering with the execution of their responsibilities. Given the possibility of facing high-risk situations, the detection and exclusion of any potentially incapacitating condition is critical to preserve operational safety and mission integrity.^[2]^

It should be noted that there are certain common dental conditions that may temporarily incapacitate the individual, posing a serious potential risk to other personnel deployed with them.^[3]^

These minimum health requirements are determined by the General Health Inspectorate, establishing scales inspired by those required by North Atlantic Treaty Organization for its members. In deployments in areas of operations, a large part of the health care that must be provided to deployed military personnel is dental care, with dental caries being the main pathology involved, either directly or indirectly (problems with fillings performed).^[1]^

This is the ultimate objective of military health care. Firstly, prevention and, secondly, rapid and effective treatment to recover the individual’s operability and minimize the risk of suffering any kind of disabling pathology during the exercise of the missions entrusted.^[2]^

For all these reasons, it is important to know epidemiological data on the prevalence of certain potentially disabling pathologies, as well as the need for certain treatments in the military population in order to establish the appropriate preventive and/or therapeutic measures, and also to effectively plan the approach to the management of these entities, establishing the human and material resources necessary to achieve the objective.

One of the branches of military dentistry is called Expert Dentistry, where the dental examination of all military personnel is contemplated in order to determine the dental aptitude of personnel to be deployed in Operational Territory or Zone of Operations and personnel commissioned to services involving prolonged isolation situations (naval voyages, services on the Islands and Rock of North Africa, commissions in the Antarctic.).^[3–5]^ The General Inspectorate of Defence Health (IGESANDEF) established a protocol in 2006 detailing the procedure for carrying out the dental examination and filling in the Dental Record.

The IGESANDEF established a protocol in 2006 detailing the procedure for the examination and completion of the Dental Record,^[6]^ one of its main functions being to record the oral and dental condition of the personnel examined and, according to this condition, to determine their suitability or lack of suitability for the performance of certain activities or deployments.

The criteria followed by IGESANDEF are as follows^[7]^:

“Military personnel whose oral condition does not require assistance or is unlikely to require emergency dental treatment in the next twelve months will be considered fit. Temporary unfitness will be determined when it is foreseen that the dental condition may prevent a normal life, in terms of nutrition and the possibility of toothache.”

This definition, being so generic, leaves it up to the dentist to determine suitability or not based on somewhat imprecise parameters. It is not easy to determine whether a certain pathology will require urgent treatment in the next twelve months. The dentist’s experience can be decisive in this respect and “help” him or her to make the right judgement.

In 2014, the IGESANDEF in its Technical Instruction Nº3 established a list of possible causes for a loss of dental aptitude, in line with the aptitude criteria established by North Atlantic Treaty Organization for member countries (Table 1) These are: activity of caries; pulp lesions; acute or uncontrolled periodontal disease; insufficiently retained or stable dentures; presence of root debris or included teeth; presence of fistulae; and episodes of recurrent pericoronitis.^[8,9]^ It is worth noting that certain conditions may temporarily result in a classification of unfit, yet can be easily resolved following a simple visit to the dentist – after which individuals may be reevaluated as fit. Therefore, it is always recommended that a dental consultation be undertaken prior to the final assessment, in order to address such readily treatable issues.

**Table 1:** Causes of loss of dental fitness according to IGESANDEF it no. 3 (2014).

Cause of loss of dental fitness	Description/ Remarks
Active caries with cavitation	Affecting dentin
Pulp lesions	With signs and symptoms of reversibility
Periodontal disease	Loss of bone/mobility
Insufficiently retained or stable dentures.	That do not ensure functionality or stability
Presence of root debris or included teeth	When there is clinical or radiological evidence of pathology
Presence of fistulae	Irrespective of origin or condition
Episodes of recurrent pericoronitis	Repeated inflammation of the tissue around a partially erupted tooth (usually wisdom teeth)

IGESANDEF = General Inspectorate of Defence Health.

There are numerous epidemiological studies focusing on the general population; however, those focusing specifically on the military population are considerably less frequent. Most of the studies aimed at this group have been carried out by military personnel, which introduces a particular focus in the approach of the objectives, mainly aimed at guaranteeing the operability of the personnel in the exercise of their functions. Among these studies, in chronological order, we have those of Morán, Gayo, Carroquino, Mombiedro, García and Tello.^[10–15]^ The aim of this study was to evaluate the prevalence, habits and needs of treatment of this military population and its relation with the fitness to develop activities abroad.

## 2. Material and methods

### 2.1. Study type and design

This is a cross-sectional observational study, aimed at evaluating the state of oral health and oral hygiene habits in a sample of military personnel stationed in Ceuta, with the objective of identifying therapeutic needs and factors associated with the loss of operational aptitude.

The minimum health conditions in the sense of fitness for service are determined by the highest body that manages all military health in Spain, the IGESANDEF.^[16]^

### 2.2. Selection of the sample

The study sample is made up of 691 military personnel assigned to different units of the Spanish Army that make up the Discontinuous Base “Teniente Ruiz,” in Ceuta. The reference population is made up of approximately 2800 troops. According to the sample calculation for a confidence level of 95% and a margin of error of 5%, at least 339 subjects would be required, so that the sample size achieved guarantees the representativeness and statistical validity of the results.

**Figure 1.**
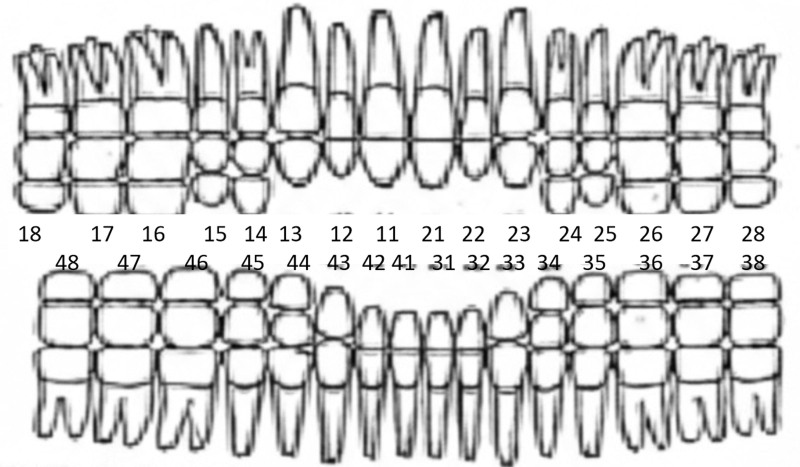
Dental factsheet used.

The selection was carried out completely randomly, with the lists of personnel to be studied being drawn up by the Personnel Sections (S1) of each of the units, taking advantage of the fact that it is compulsory to update each individual’s Dental Record annually^[17]^ with an standardized factsheet (Fig. 1).

### 2.3. Data collection

The study begins in October 2019 and ends in March 2020.

The examinations were carried out in the Dental Office of the “Teniente Ruiz” Discontinuous Services Unit (USBAD “Teniente Ruiz”) in Ceuta following a Dunning type 3 study model: inspection using a mouth mirror, explorer and periodontal probe type World Health Organization with adequate lighting.^[17]^

A single examiner (A.B.B.) with an intraexaminer kappa index of 0.92 carried out both the dental examination and the completion of the questionnaire, to ensure perfect understanding of the questions posed.

The epidemiological indicators studied were: caries prevalence; decayed, missing, and filled teeth (DMFT) and restoration index; community periodontal index; attachment loss and prevalence of periodontal disease.

### 2.4. Socio-demographic variables of the study

In order to carry out the study, socio-demographic data were collected in relation to: date of birth and age (dividing the sample into 3 groups: 20 to 34; 35 to 44 and 45 to 60 years old); sex (M/F); Levels of study (basic, intermediate and higher); geographical location of origin (urban, peri-urban or rural); military employment (Commanders, Non-Commissioned Officers and troops); unit of destination (high operability or medium operability); and cultural/geographical group (Peninsular Spanish and North African of European origin, North African Spanish of non-European origin, Spanish of Central African, European, Hispanic-American and other origin).

### 2.5. Oral health variables of the study (numbered in Table 2)

#### 2.5.1. Ethical considerations of the study

Authorization was obtained from the Chief General of the Teniente Ruiz Discontinuous Base to carry out this study by means of a reasoned request using the Ministry of Defence corporate email address. The study was covered by the Ethics committee of Odontologia Social Foundation of Seville (Spain) with the number 03/19 approved in September of 2019. At the time of the dental examination of the personnel, they were informed of the study to be carried out and their consent was obtained to include their data in the study.

**Table 2:** Oral health variables.

Variable	Categories/ Observed details
Habits	Smoking- Alcohol consumption- Oral hygiene
Prosthetic need	Total- Partial (excludes purely aesthetic indications)
Type of prosthesis	Complete removable- Partial removable- Crowns- Bridges- Implants
Patient’s perception	No need- Functional need- Aesthetic need- Pain-related need
Examiner’s assessment	Good oral health- Requires treatment
Mucosal pathology	List of lesions (e.g., candidiasis, leukoplakia, ulcers)- Specific locations
TMJ examination	Joint noises- Pain- Mandibular deviation- Mouth opening (in mm)
Eruptive pathology	Wisdom teeth- Other unerupted teeth- Recent discomfort
Military fitness criteria	Fit- Temporarily unfit (according to official guidelines)
Dental condition and treatment	Global diagnosis- Therapeutic needs (restorative, surgical, preventive, etc)

## 3. Results

The population sample analyzed in this study consisted of a total of 691 active-duty military personnel stationed at the Teniente Ruiz Discontinuous Base in Ceuta. The distribution of the sample by gender, age, rank, place of origin, assigned unit, and educational level is presented in Table 3.

**Table 3:** Sociodemographic frequency table (n = 691).

Variable	Categories	Frequency (n)	Percentage (%)
Gender	Female	61	8.83
	Male	630	91.17
Place of origin	Urban	174	25.18
	Peri-urban	415	60.06
	Rural	102	14.76
Age group	20–34	342	49.49
	35–44	251	36.32
	45–60	98	14.18
Deployment level	High operations	391	56.58
	Medium operations	300	43.42
Rank	Officer	198	28.65
	Troop	493	71.35
Education level	Basic	144	20.84
	Intermediate	425	61.51
	High	122	17.66
Total	–	691	100.00

**Figure 2.**
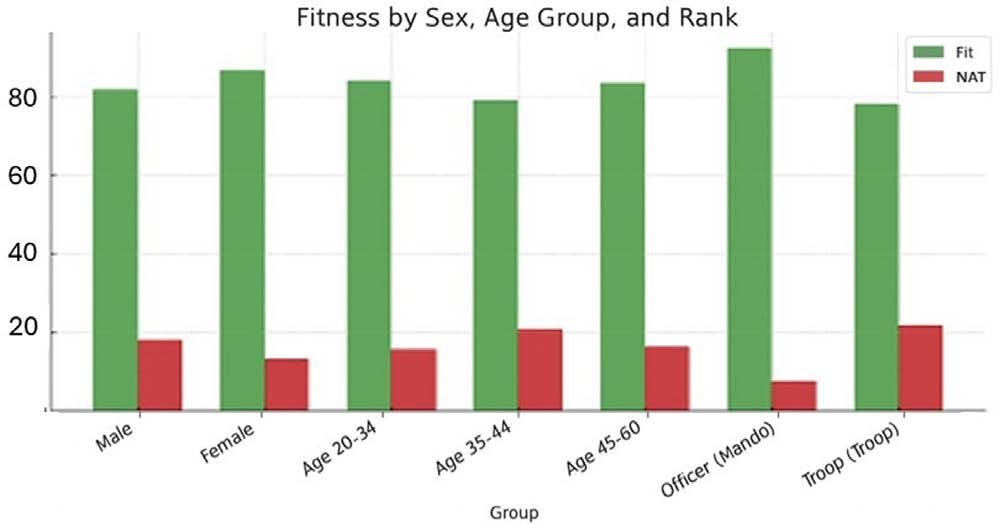
Distribution of fitness status by group.

Table 4 and Figure 2 shows the fitness percentages of the sample in relation to gender, age and employment. A strong statistically significant association was found with employment (*P* < .000), with the troop class having the highest percentages of temporary unfitness. The variables sex and age do not appear to be related to fitness (*P* > .05). In terms of data presentation, we have found in this study that men have higher percentages of NAT than women. This is also the case in the 35 to 44 age group.

**Table 4:** Percentage of fit and NAT individuals by group.

Group	Fit (%)	NAT (%)
Male	81.9	18.1
Female	86.89	13.11
Age 20–34	84.21	15.79
Age 35–44	79.28	20.72
Age 45–60	83.67	16.33
Officer	92.42	7.58
Troop	78.3	21.7

NAT = temporarily unfit.

### 3.1. Analysis of aptitude in relation to habits: tobacco, alcohol, oral hygiene

In our sample we have 122 individuals (17.66%) who are temporarily unfit (NAT) according to military criteria. Most of them are concentrated in the troop class (21.7% vs 7.58%).

**Figure 3.**
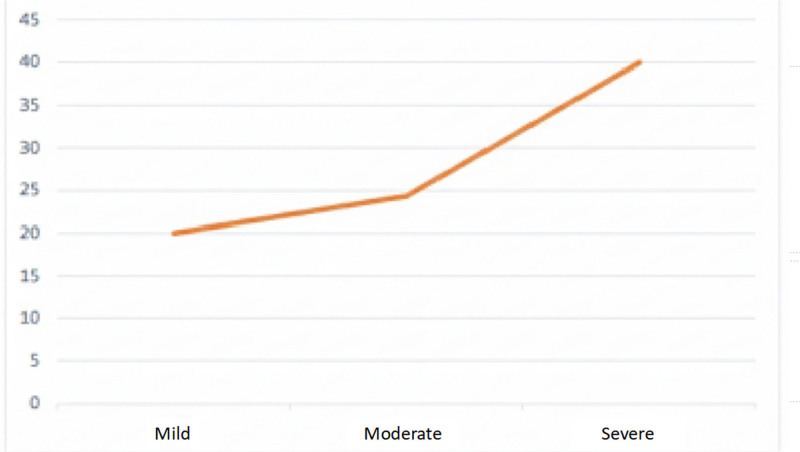
Distribution of NAT by smoking frequency (*P* = .000). NAT = temporarily unfit.

As shown in Table 5, all aspects related to smoking are associated with a higher prevalence of unfitness. Statistical significance is strong in all smoking-related aspects, so that nonsmokers have higher percentages of fitness than smokers/ex-smokers (*P* < .000). Furthermore, as the frequency of light to moderate to severe smoking increases, the NAT percentages tend to increase significantly (*P* < .000) (Fig. 3).

**Table 5:** Attitudes towards habits.

	Fit	NAT	*P*
Tabaquism			
Smoker	74.06	25.94	<.000
Non smoker	86.01	13.99	
Alcohol			
Habitual	66.67	33.33	
Occasional	83.78	16.22	
No alcohol	80.95	19.05	
Hygienic habits			
No use	66.67	33.33	
Dayly	82.55	17.45	
No interproximal brush	81.73	18.27	>.05
Interproximal brush	88.06	11.94	
No floss	80.63	19.37	<.05
Use floss	88.31	11.69	
No rinse	85.93	14.07	<.05
Use rinse	79.12	20.88	

This is not the case for alcohol consumption, where it was not possible to find a statistical difference because the sample did not meet the requirements.

There is a correspondence between frequent use of oral hygiene utensils and higher prevalence of aptitude. Regarding toothbrush use and aptitude, we were unable to test the hypothesis due to the small sample size in some aspects, but the highest percentages of lack of aptitude are found in subjects who do not brush.

Regarding interdental brushing and proficiency, we found no statistical significance (*P* > .05). The percentages of NAT are similar between users and non-users of interdental brushes.

Flossing, on the other hand, is significantly associated with an improvement in proficiency rates (*P* < .05).

When we analyzed the data related to the use of mouthwash and proficiency, we found a statistical association for both use and frequency of use (*P* < .05). The statistical association is along the lines that the use of mouthwash is associated with a higher percentage of NAT.

### 3.2. Analysis of fitness in relation to different oral health variables

Table 6 shows the data obtained for fitness according to different oral health variables.

**Table 6:** Results of the aptitude related to oral health.

Category		Fit	NAT	*P*
DMFT	DMFT≤3	95.93	4.07	.000
	CMFT > 4	79.40	20.60	
Caries component	C = 0	94.52	5.48	.000
	C=>1	68.71	31.29	
Treatment needs and prosthesis	Preventive	76.72	23.28	.001
	Conservatory	69.18	30.82	.000
	Endodontics	21.05	78.95	
	Extraction	20.69	79.31	.000
	Other	20.69	79.31	.000
	Prosthesis	71.61	28.39	.000
CPI (1 or more affected sextants)	CPI = 0 (healthy)	84.89	15.11	.000
	CPI = 1 (bleeding)	73.08	26.92	.000
	CPI = 2 (calculus)	71.43	28.57	.000
	CPI = 3 (depth 4–5 mm)	*68.97*	*31.03*	.054
Loss of insertion	0–5 mm	83.49	16.51	.000
	6–11 mm	56.25	43.75	
Subjective perception	Treatment needed	64.03	35.97	.000
	No need	96.65	3.35	

CPI = community periodontal index, DMFT = decayed, missing, and filled teeth, NAT = temporarily unfit.

#### 3.2.1. DMFT index and decayed component (D)

In our sample, all individuals classified as NAT have a DMFT index >0.

We found that for a DMFT index value <4, the percentage of NAT is 4.07%, but once the index exceeds 4, the percentage of NAT multiplies by 5. The statistical association is strong with *P* = .000.

It is evident that the tendency for an increasing percentage of NAT according to the DMFT index value is due to the pathological component included in this index.

The decayed component (D) of the DMFT index, which is indicative of caries, is strongly and significantly associated with a higher percentage of NAT, multiplying by 6 the percentage of NAT compared to those without caries (*P* = .000).

#### 3.2.2. Treatment needs and prosthetics

The data indicate a significant association between the need for treatments and a greater loss of fitness. This significance occurs for the need for preventive, conservative, extraction, prosthetic, and other types of treatments (*P* < .05).

**Figure 4.**
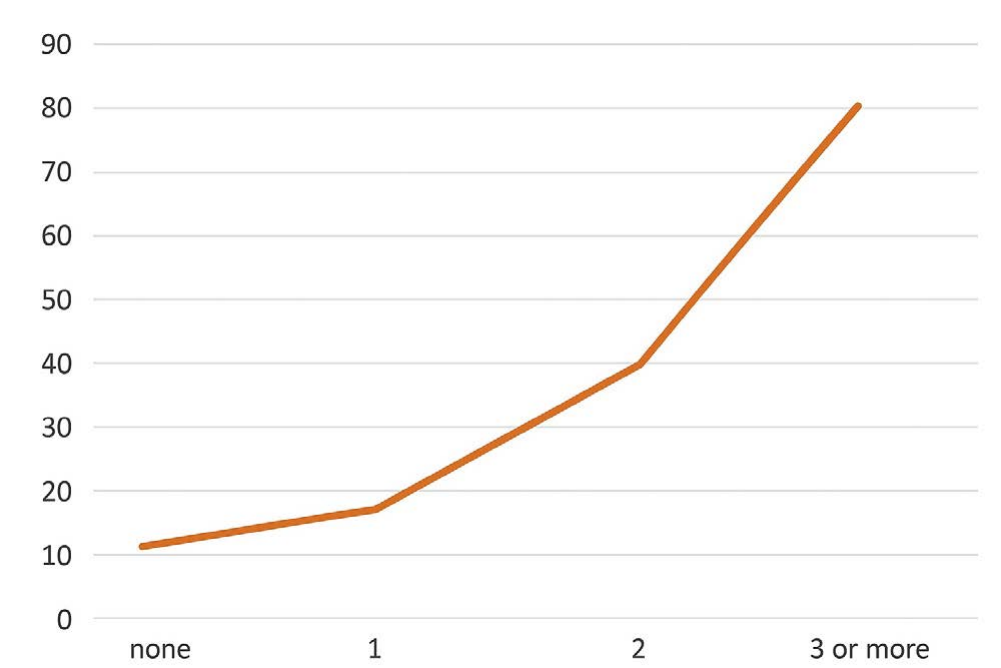
Distribution of NAT according to the need for multiple treatments (*P* = .000). NAT = temporarily unfit.

When analyzing multiple treatment needs – that is, fitness in relation to the need for more than one different treatment – we find that the higher the number of treatments needed, the higher the percentage of temporary unfitness. There is a clear upward trend in this regard, with strong statistical significance (*P* < .000) (see Fig. 4).

#### 3.2.3. Regarding the community periodontal index

When analyzing fitness according to the number of sextants affected by bleeding, calculus, or pockets, we found that when one or more sextants are affected, the percentage of temporary unfitness increases compared to the percentage found in healthy sextants. The statistical association is strong (*P* = .000), except in the case of PI = 3, where significance was not reached, possibly due to sample size (*P* = .054). This clearly shows the influence of periodontal disease on the loss of fitness.

#### 3.2.4. Regarding the periodontal insertion loss

Greater loss of fitness is significantly associated with a higher presence of elevated levels of insertion loss. The statistical association is strong (*P* = .000).

#### 3.2.5. Regarding subjective perception

Our results indicate that there is coherence between what subjects perceive about their health status and the fitness determination obtained. We found a strong statistical association (*P* = .000) supporting this statement.

**Figure 5.**
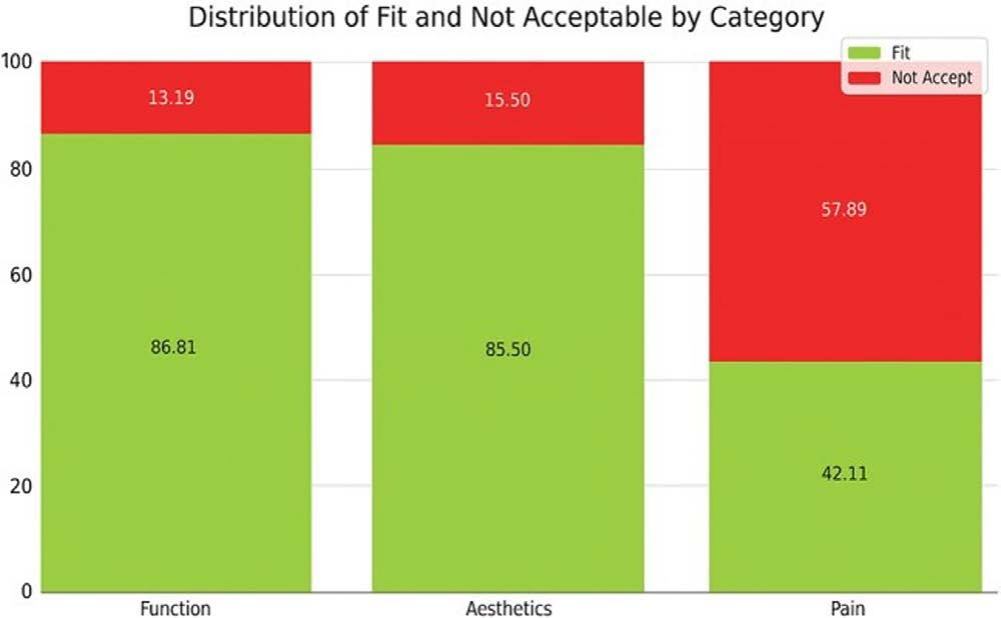
Distribution of fitness according to perceived type of required treatment.

Regarding the reason why subjects perceive the need for treatment, pain is associated with the highest percentages of NAT, followed by aesthetics, and lastly, functional rehabilitation. Again, the statistical association is strong (*P* = .000) (see Fig. 5).

#### 3.2.6. Regarding the professional assessment

**Figure 6.**
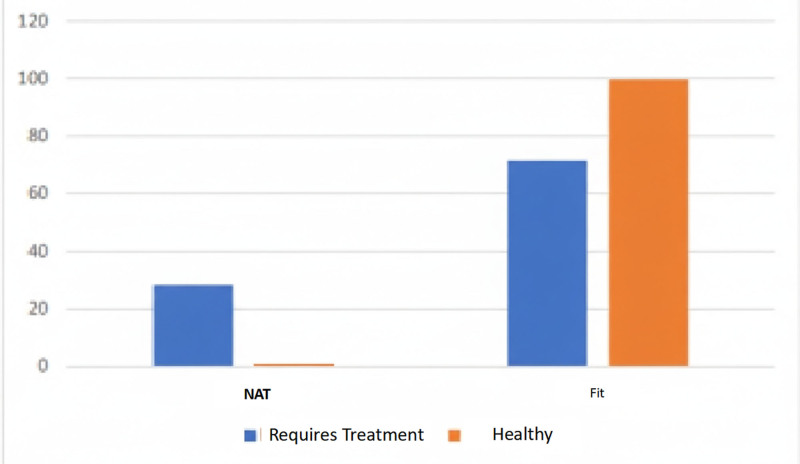
Distribution of military fitness according to professional assessment.

The professional’s assessment of the subjects’ treatment needs is consistent with the results obtained. Only 0.38% of those considered healthy were classified as NAT. The hypothesis test could not be performed because some necessary requirements were not met (see Fig. 6).

#### 3.2.7. Regarding eruptive pathology

**Figure 7.**
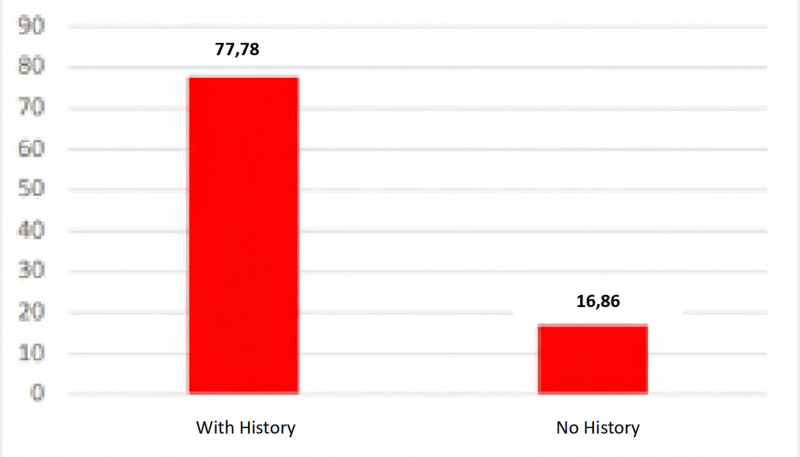
Distribution of NAT in partially erupted third molars. NAT = temporarily unfit.

This is determined by the existence of a prior history of repeated discomfort caused by a partially erupted third molar. The results we obtained when comparing fitness and the existence of this history of discomfort indicate that a high percentage of temporary unfitness is associated with this condition. These results are not supported by a hypothesis test, as it was not possible to perform one due to the sample being non-representative (see Fig. 7).

#### 3.2.8. Regarding joint pathology

**Figure 8.**
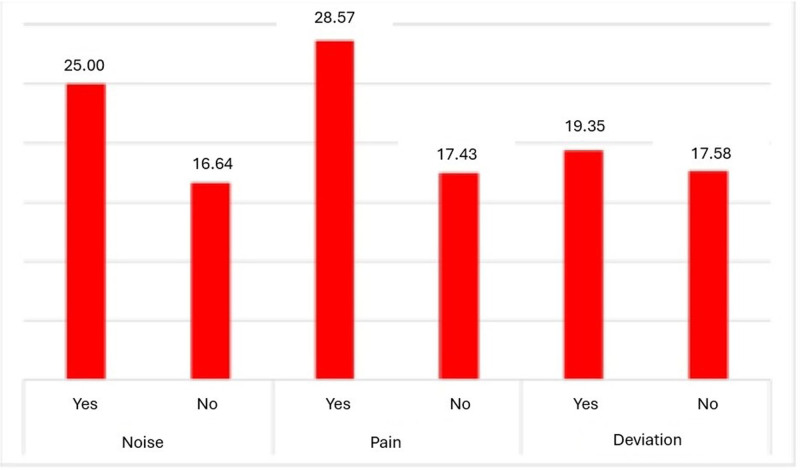
Distribution of % NAT according to TMJ signs (*P* > .05). NAT = temporarily unfit.

Our results indicate that the presence of signs indicative of joint pathology (sounds, pain, and deviation) are associated with higher percentages of temporary unfitness. In the cases where hypothesis testing was possible, no statistical significance was found. The data are shown in Figure 8.

#### 3.2.9. Regarding mucosal pathology

In our sample, we found an association between the presence of lesions and the maintenance of fitness. It is important to note that the lesions found were benign in nature and occurred in a small percentage (3.47%), so they do not affect the determination of loss of fitness in any way.

## 4. Discussion

There are many studies that analyze the prevalence of dental emergencies in deployment situations.

To facilitate comparison of our results with those of other similar studies, the methodological recommendations proposed in the World Health Organization publication “Oral Health Surveys. Basic Methods” (5th Ed.), for conducting epidemiological studies of oral health.^[17]^

The determination of fitness in the Armed Forces is a procedure that follows the criteria indicated in the various military health regulations,^[7,9,18–20]^ which aim to maintain optimum levels of operability and whose objective is to return the person concerned to his or her duties as soon as possible, maintaining su salud y reduciendo los efectos negativos de la patología en su operatividad.^[21]^

In our study, 17.66% (n = 122) were NAT. This is a small figure considering other studies on the Spanish military population, such as that of Tello, who obtained 30% NAT. This same author, 9 years later, obtained much lower NAT data, just over 8%.^[15]^ Considering the same population, the improvement is remarkable. The success of the preventive programmes implemented after the first screening seems to be responsible for this improvement.^[22–24]^ It should also be acknowledged that, in 2014, the criteria were revised – though this does not necessarily imply that the new standards were either more or less stringent than the previous ones. It is our view that the intention was to formalize, through a written document, the acceptance criteria for military operations.

There is no doubt that the regular checkups to which the military health service subjects soldiers are an element that has an influence on improving the health of the group.^[13]^ Troop rank and male sex are variables that predominate in the NATs as a whole. The same results were obtained by Tello in his study.^[15]^

No studies have been found that directly relate the outcomes of periodic dental examinations with the determination of fitness for duty. This information is crucial, as identifying the presence of dental pathologies that may cause emergencies during a potential deployment allows for timely preventive or therapeutic actions to mitigate such risks^[25–27]^

By acting on these findings, we can potentially reverse high-risk classifications (Class 3 or 4) and reclassify individuals as Class 1 or 2, making them fit for deployment. Despite having the tools available for such interventions, studies such as that of York et al demonstrated that only 57.4% of those initially classified as Class 3 were reclassified as Class 1 within a four-year period.^[28]^

In our fitness analysis, we found that smoking is strongly associated with the percentages of Temporary Unfitness, both in terms of smoking status and frequency of the habit. Zajc and colleagues studied the effect of smoking in Croatian military personnel, finding that smokers presented higher rates of periodontal problems and greater loss of operability than nonsmokers.^[29]^ In response to the high prevalence of smoking, targeted strategies should be incorporated to mitigate its impact as a preventable cause, with an emphasis on early detection, patient education, and pre-assessment interventions.

As we have seen, the same does not apply to alcohol. Although data indicate that in habitual drinkers, the prevalence of NAT is much higher. Following military fitness criteria, it seems obvious that when there is a manifest need for treatment, the likelihood of being classified as NAT increases. Our study supports this statement. A statistically significant association was found between the variable “need for treatment” and the variable “fitness,” in almost all treatment modalities, except for endodontic treatment, which could not be analyzed due to its small sample size. This finding is reflected in studies like that of Skec and colleagues, which report a prevalence of dental emergencies in military personnel classified as Class 3 of 66.2%^[30]^

When analyzing data related to mouthwash use and fitness, a statistical association was found both for use and frequency of use (*P* < .05). This statistical association aligns with the finding that mouthwash use is associated with a higher percentage of NAT. Analyzing this clinical inconsistency, we concluded that the only explanation is improper use of mouthwash – used as a substitute for brushing rather than as a complement to it. In our clinical experience, it is not uncommon to have to instruct patients on this issue.

Our results indicate a strong association between caries prevalence and the percentage of NAT. A DMFT index > 3 corresponds to a 20.6% percentage of NAT in our sample. This is 5 times the percentage associated with a DMFT ≤ 3. The importance of caries is confirmed as a determining factor in military fitness. Most authors studying dental emergencies during deployments agree that a high percentage of these are due to this pathology.^[31,32]^

In this regard, Richardson quantified caries by the amount of dentin remaining to the pulp, classifying military personnel as Class 2 or Class 3 depending on whether <2 mm (Class 3) or equal/>2 mm (Class 2) remained.^[33]^

When analyzing data related to eruptive pathology, it should be noted that the mere presence of one or more partially erupted third molars does not imply loss of fitness. Although eruptive pathology was not very prevalent in our study, it is responsible for a large number of emergency visits during deployments.^[20,22,23,25]^ This pathology leaves open the debate on whether and under what criteria partially erupted third molars should be extracted before deployment.^[34,35]^ An observable factor is the presence of malpositioned upper third molars, which may contribute to the onset of pericoronitis in the lower third molar region. Although this relationship is often noted clinically, it remains underreported in the literature and warrants further investigation within such cohorts. Where available, data regarding the presence of buccoverted or ectopically positioned upper third molars – particularly in individuals deemed fit at deployment but who subsequently developed symptoms – could be valuable. If asymptomatic third molars are not currently grounds for a “Temporarily Not Fit” classification, then the presence of upper third molars in atypical orientations or eruptive patterns may merit consideration as an additional criterion for future updates to the guidelines.^[36]^

On another note, it would be valuable to analyze the sensitivity and specificity of the criteria set forth in 2014 for assessing fitness or unfitness. This would allow for the evaluation of false positives and false negatives in this assessment, thereby subjecting the evaluation method to a thorough and appropriate validation.

## 5. Conclusions

Of the total number of personnel assessed for military missions abroad, 17.66% were classified as NAT due to dental problems. This percentage represents a significant number of personnel who cannot be deployed, which has direct consequences on both operational planning and human resource availability. Dental caries was identified as the main determinant of loss of fitness to deploy.

Eruptive pathology (such as eruption of wisdom teeth) was also identified as a predictable problem, which underlines the importance of adequate and long-term dental planning. In this context, it becomes evident that it is crucial to implement secondary prevention measures in National Territory, the application of which would significantly reduce the prevalence of dental emergencies in the Area of Operations, contributing to the stability and continuity of missions abroad.

The military dentist plays a strategic role and must take a proactive approach. This implies an in-depth knowledge of the epidemiological profiles and risk factors of their target population in order to adapt their clinical and preventive activities efficiently.

## Author contributions

**Conceptualization:** Alejandro Bel-Blesa, Javier Flores-Fraile, Eva Rosel-Gallardo, David Ribas-Pérez.

**Data curation:** Alejandro Bel-Blesa, Marta Hernández-Donadeu, Javier Flores-Fraile, Juan Gómez-Salgado, Julio Torrejón-Martínez, Eva Rosel-Gallardo, David Ribas-Pérez, Antonio Castaño-Séiquer.

**Formal analysis:** Javier Flores-Fraile, Antonio Castaño-Séiquer.

**Funding acquisition:** Antonio Castaño-Séiquer.

**Methodology:** Javier Flores-Fraile, Eva Rosel-Gallardo, David Ribas-Pérez.

**Project administration:** Eva Rosel-Gallardo, David Ribas-Pérez.

**Resources:** Luis El Khoury-Moreno, Antonio Castaño-Séiquer.

**Software:** Juan Gómez-Salgado, Julio Torrejón-Martínez.

**Supervision:** Juan Gómez-Salgado, Luis El Khoury-Moreno.

**Visualization:** Marta Hernández-Donadeu, David Ribas-Pérez.

**Writing – original draft:** Alejandro Bel-Blesa, Marta Hernández-Donadeu.

**Writing – review & editing:** Julio Torrejón-Martínez, David Ribas-Pérez.
